# Bacterial Community Composition in Three Freshwater Reservoirs of Different Alkalinity and Trophic Status

**DOI:** 10.1371/journal.pone.0116145

**Published:** 2014-12-26

**Authors:** Marc Llirós, Özgül Inceoğlu, Tamara García-Armisen, Adriana Anzil, Bruno Leporcq, Lise-Marie Pigneur, Laurent Viroux, François Darchambeau, Jean-Pierre Descy, Pierre Servais

**Affiliations:** 1 Laboratory of Freshwater Ecology, University of Namur, Namur, Belgium; 2 Ecologie des Systèmes Aquatiques, Université Libre de Bruxelles, Brussels, Belgium; 3 Chemical Oceanography Unit, University of Liège, Liège, Belgium,; Institute of Tibetan Plateau Research, China

## Abstract

In order to investigate the factors controlling the bacterial community composition (BCC) in reservoirs, we sampled three freshwater reservoirs with contrasted physical and chemical characteristics and trophic status. The BCC was analysed by 16S rRNA gene amplicon 454 pyrosequencing. In parallel, a complete dataset of environmental parameters and phytoplankton community composition was also collected. BCC in the analysed reservoirs resembled that of epilimnetic waters of natural freshwater lakes with presence of *Actinobacteria*, *Alpha- and Betaproteobacteria*, *Cytophaga–Flavobacteria–Bacteroidetes* (CFB) and *Verrucomicrobia* groups. Our results evidenced that the retrieved BCC in the analysed reservoirs was strongly influenced by pH, alkalinity and organic carbon content, whereas comparatively little change was observed among layers in stratified conditions.

## Introduction

Microbes play a central role in global environmental processes and earth biogeochemistry [1], with bacteria being the most important component of microbial communities responsible, in aquatic ecosystems, for the organic matter mineralization and nutrient recycling processes [2], [3]. However, due to the intrinsic complexity of bacterial diversity and the small fraction of bacteria that can be cultivated, knowledge on the driving factors of bacterial community composition (BCC) has remained elusive until the last decades. In this sense, the introduction and wide use of molecular tools in microbial ecology has rapidly increased the knowledge on bacterial diversity, identity and involvement in key processes over many different environments [4]–[8]. As a consequence, microbial ecologists have been looking for drivers of the BCC fluctuations in relation with the functioning and the ecology of aquatic ecosystems.

Freshwater environments have been traditionally studied focusing on their physical and chemical characteristics and community composition, from algal assemblages to fish, and a considerable body of literature has been devoted to the ecology of these communities. In a context of environmental management, shifts in community composition have been used as indicative of shifts in the water bodies resulting from variation of both internal (i.e., physical and chemical properties of the water column or retention time) and external (i.e., climate conditions, organic matter and nutrient inputs) processes [9]–[13]. Concerning the microbial communities, it has been shown that abiotic (e.g., temperature, oxygen, pH, conductivity, water transparency, organic matter concentration and biodegradability, nutrients, and damming) as well as biotic (chlorophyll (Chl) *a* content, interactions with phyto- and zooplankton, grazing, competition) factors regulate temporal and spatial shifts of microbial communities in aquatic environments [9], [14]–[23].

Today, deep sequencing technologies such as 454 pyrosequencing provide an attractive avenue to explore complex microbial communities [4], [5], [24], [25] and evidenced the existence of a “rare biosphere” [26]–[28]. Moreover, ubiquitously distributed uncultured bacterial phylotypes have been also reported in freshwater lakes [29]–[33]. Newton and co-workers [34] reviewed the BCC present in epilimnetic waters of lakes worldwide, showing that over the most recovered phyla are: *Proteobacteria*, *Actinobacteria*, *Cytophaga–Flavobacterium–Bacteroidetes* (*CFB*), *Cyanobacteria* and *Verrucomicrobia*. In fact, *Betaproteobacteria* are by far the most studied and often the most abundant (up to 60 – 70% of total cells) bacteria in epilimnetic waters [34]. This bacterial group is mainly driven by high DOC and nitrate concentrations, and often appears associated with cyanobacteria or particles of different sizes [23], [35]–[38]. Nevertheless, most of the studies included in Newton and co-workers' review [34] were only based on surface or epilimnetic waters surveys from natural freshwater lakes or lagoons.

In spite of the knowledge recently gained on BCC and their ecological drivers in natural freshwater environments, there is a lack of knowledge on man-made reservoirs, with some exceptions [14], [20], [39]–[41]. Freshwater reservoirs present some specificities in comparison to natural lakes resulting in different biological, physical and chemical characteristics, such as shorter retention times, water level fluctuations, important shifts of nutrient and dissolved organic carbon (DOC) concentrations and scarcity of littoral macrophytes which all together affect the planktonic organisms present [20], [41]–[43].

In an attempt to identify the main drivers of the bacterial assemblage developing in freshwater reservoirs, we analysed three water bodies with contrasting physical and chemical characteristics, trophic status, DOC content and phytoplankton assemblage at three distinct periods of the year (spring, summer and fall). We hypothesised that those physical and chemical characteristics like pH, organic carbon content, or phytoplankton community composition may exert some influence on BCC in freshwater reservoirs.

## Material and Methods

### Sampling sites and sample collection

Three freshwater reservoirs located in Belgium (see Table 1 and S1 Fig. for exact locations) were sampled in April, July and October 2010. These reservoirs, named La Gileppe, Ry de Rome and Féronval, were selected in order to obtain contrasting physical, chemical, and trophic conditions. The characteristics of the studied reservoirs were described in [44] and classified according to the European classification of lakes [45]. La Gileppe reservoir is a deep (maximum depth 58 m), oligotrophic, cold-water body covering 130 ha, with a catchment area dominated by forests of conifers and deciduous trees. The pH of its waters is low, related to the geology of the watershed and the presence of peatbogs. It has a mean annual residence time of 315 days and belongs to the Central Baltic lake type (L–CB3; lowland, shallow lakes with an alkalinity between 0.2 and 1.0 meq L^−1^) albeit with lower pH and greater depth than the range of this lake type. These characteristics make it actually closer to Northern humic lakes of very low alkalinity. Ry de Rome reservoir is an oligo-/mesotrophic water body of 26 ha of surface area and a maximum depth of 25 m. It has been also classified as L–CB3 [44] and has a mean retention time of 200 days. The third reservoir (Féronval) belongs to the L–CB2 lake type (lowland, very shallow, calcareous, alkalinity> 0.5 meq L^−1^) with a surface area of 21 ha and a maximum depth of 12 m (mean depth 3.8 m). Féronval is a poly-eutrophic reservoir; with mean total phosphorus (TP) concentration of 55 µg L^−1^ and Chl *a* maxima higher than 100 µg L^−1^.

**Table 1:** General characteristics of the three analysed reservoirs and annual average values of some physical and chemical parameters.

Parameters^a^	La Gileppe	Ry de Rome	Féronval
Latitude (N)	50° 35′ 16.73′′	50° 01′ 21.67′′	50° 12′ 59.14′′
Longitude (E)	5° 58′ 34.75′′	4° 32′ 13.55′′	4° 23′ 25.34′′
Altitude (m)	310	280	220
Surface of basin (km^2^)	54	10	10
Volume (hm^3^)	26.4	2.2	0.8
Surface (ha)	130	26	21
Retention time (years)	0.86	0.55	0.31
Max depth (m)	58	25	12
Mean depth (m)	20.3	8.5	3.8
Zeu (m)	3.60	9.89	3.49
Zm (m)	5.67	4.33	5.33
Zm/Zeu	1.53	0.49	1.78
pH	5.71	6.81	7.86
Conductivity (μS cm^−1^ at 25°C)	57	59	392
DO (mg O_2_ L^−1^)	9.34	8.64	8.97
Turbidity (NTU)	3.57	1.90	6.79
TAC (meq L^−1^)	0.50	0.87	13.14
DIN (mg L^−1^)	0.63	0.72	2.21
SRP (mg L^−1^)	0.003	0.003	0.005
TP (mg L^−1^)	7.33	5.00	55.67
Si (mg L^−1^)	2.08	2.14	1.52
BOD_5_ (mg O_2_ L^−1^)	1.0	1.4	1.4
COD (mg O_2_ L^−1^)	19.0	5.0	11.9
DOC (mg C L^−1^)	6.74	2.66	4.00
TOC (mg C L^−1^)	8.39	3.12	5.50
Alkalinity (meq L^−1^)^b^	0.2	0.21	2.64
Chlorophyll *a* (μg L^−1^)	0.5	2.62	13.44

Values correspond to the average of six depths sampled in each reservoir, each month between March and October 2010.

a , Zeu: Depth of euphotic zone, Zm: Depth of mixing zone, Cond: conductivity, TAC: total alkalinity, DIN: dissolved inorganic nitrogen, SRP: soluble reactive phosphate, TP: total phosphorus, Si: dissolved silica, BOD_5_: biological oxygen demand, COD: chemical oxygen demand, DOC: dissolved organic carbon, TOC: total organic carbon; ^b^, [44].

During the three sampling campaigns, water samples representing the three main water compartments (i.e., epilimnion, metalimnion and hypolimnion, when the reservoirs were stratified) were collected in each reservoir using a 5.0 L Niskin bottle. Water samples for chemical and biological analyses were kept in a cooler until further analyses in the laboratory on the day of sampling. In all cases, the analysed reservoirs did not involve endangered or protected species thus no specific permissions were required for sample collection.

### Physical and chemical analyses

The water column structure in the three reservoirs was determined using a multiparameter probe YSI 6000 V2 (Yellow Spring Instruments, USA) to determine vertical depth profiles of temperature (T), pH, conductivity (Cond), turbidity (Turb) and dissolved oxygen (DO). The depth of the mixed layer (Zm) was estimated from T and DO vertical profiles.

In addition, a large set of physical and chemical data was obtained from monthly sampling campaigns collecting water samples from six representative water depths, carried out by the ISSeP (Institut Scientifique de Service Public) from a water quality survey program in the context of a Water Framework Directive monitoring program. These data included Secchi disk depth measurements, TAC (total alkalinity), DIN (dissolved inorganic nitrogen; i.e., the sum of ammonium, nitrite, and nitrate concentrations), SRP (soluble reactive phosphate), TP (total phosphorous), Si (dissolved reactive silica), BOD_5_ (biological oxygen demand) and COD (chemical oxygen demand). All analyses were carried out following standard methods [46].

For dissolved organic carbon (DOC) analyses, water samples were filtered on GF/F glass fiber membranes (Whatman) previously combusted at 550°C. Glassware receiving water samples for DOC analyses were previously muffled at 550°C for 4 h after cleaning. Sample preservation was done by the addition of NaNO_3_ (0.05%, final concentration). DOC was measured after removal of inorganic carbon by bubbling in the presence of phosphoric acid using a total organic carbon analyser (Dohrman Apollo 2000, Dohrman) in which organic carbon was oxidised at high temperature and CO_2_ produced was detected by infrared spectrometry. The same procedure was carried out on unfiltered samples for total organic carbon (TOC) determination.

### Phytoplankton pigment analyses

Samples for Chl *a* and secondary pigment analyses were treated following a procedure previously described [47] using High-Performance Liquid Chromatography (HPLC) with a Waters system comprising a NovaPak C18 HPLC column, a Waters 996 PDA detector and a Waters 470 fluorescence detector. Calibration was made using commercial external standards (DHI, Denmark).

### Bacterial abundance and production

Water samples for bacterial abundance estimation were passed through 10.0 µm pore–size 47 mm diameter cellulose acetate filters (Whatman) and preserved by the addition of filtered (0.22 µm MILLEX) formaldehyde (2% final concentration) and stored at 4°C until analysis. Fixed water samples were subsequently passed through 0.22 µm pore-size black filters (Nucleopore) to retain free-living bacteria for enumeration. Bacterial abundance was determined using epifluorescence microscopy after DAPI (4,6 diamidino-2-phenylindole; 2 µg mL^−1^, final concentration) staining [48]. Briefly, after filtration of 1.0 mL of fixed water sample, 600 to 1200 cells were counted per sample.

Bacterial production was estimated from tritiated thymidine (^3^H-Thy) incorporation rates [49]. Briefly, 10 mL of water were incubated in triplicates with ^3^H-Thy (45 Ci mmol^−1^; ICN Pharmaceuticals) for one to two hours in the dark at *in situ* temperature and at saturation conditions (20 nM of ^3^H-Thy, [50]). After incubation, cold trichloroacetic acid (TCA) was added (5.0%, final concentration) and each sample was subsequently passed through 0.22 µm pore-size cellulose nitrate filters (Sartorius). Radioactivity associated with the filters was estimated by liquid scintillation in a Liquid Scintillator Analyser Tri-Carb 2100TR (Packard) apparatus. Cell production was calculated from ^3^H-Thy incorporation rates using the conversion factor of 0.5 × 10^18^ cells produced per mole of ^3^H-Thy incorporated into DNA experimentally determined for freshwater environments [50]. Bacterial production values (μg C L^−1^ h^−1^) based on thymidine incorporation were obtained by multiplying cell productions by the average carbon content (CC) per bacterial cell. In order to calculate the average CC per bacterial cell, image analysis was performed using the same microscopic preparations used for DAPI enumerations to carry out automatic measurement of bacterial cell size from digital images (Nikon DMX 1200). The LUCIA G program (Laboratory Imaging, [51]) was used for estimating cell dimension of around 1000 cells per slide and the biovolume of each cell was then calculated. Biomass was estimated from the abundance and biovolume distribution using the relationship CC  =  92×V^−0.598^, relating carbon content per cell (CC; fg C cell^−1^) to biovolume (V; μm^3^) determined from the data of Simon and Azam [52].

### Bacterial community composition: nucleic acid extraction and 16S rRNA gene amplicon 454 pyrosequencing

Samples for nucleic acids extraction were sequentially passed through 10.0 µm pore–size 47 mm diameter filters (Nuclepore), to remove particulate debris and large planktonic organisms, and through 0.22 µm pore–size 47 mm diameter DURAPORE® (Millipore, USA) filters to retain free–living prokaryotes. Filters were preserved in eppendorfs containing 2.0 mL Sucrose Buffer (Tris HCl 1 M pH 8.0, EDTA 0.5 M pH 8.0, Sucrose 1.5 M) at −20°C until further analyses. Total nucleic acids were extracted from 0.22 µm pore–size filters using enzymatic and mechanical cell lysis procedures [53], [54]. Nucleic acids concentration and purity were determined using a Nanodrop ND-2000 UV-Vis spectrophotometer (Nanodrop, DE). Finally, nucleic acid extracts were kept at −20°C until further analyses.

A total of 27 samples were individually processed for bacterial massive pyrosequencing. Bacterial tag-encoded 454 FLX amplicon pyrosequencing (b-TEFAP) was performed using a 454 FLX Titanium platform (Roche) with Titanium reagents at Research and Testing Laboratory (Lubbock, TX, USA) as previously described [55], [56]. The universal bacterial primers 28f (5′-gAg TTT gAT CNT ggC TCA g) and 519r (5′-gTN TTA CNg Cgg CKg CTg) were selected to span the V1–V3 variable region of the bacterial 16S rRNA gene [57].

Retrieved pyrosequencing data was subjected to further analyses by using the open-source software package mothur (v1.24.1, [58]). Data were decompressed and sequencing errors were reduced by trimming flows (i.e., denoising) and sequences by applying the following criteria: amplicons shorter than 200 bp in length, reads containing any unresolved nucleotides and more than 8 homopolimers were removed from the pyrosequencing-derived datasets. Subsequently, we processed improved sequences from individual files. Afterwards, putative chimeras (checked by using Uchime software [59]) and putative contaminants (understood as those unclassified or misclassified sequences after comparing candidate sequences against the latest Ribosomal Database Project training set database) were removed from our data set. Once files were considered free of chimeras or contaminants, alpha- and beta-diversity analyses by OTUs and phylogenetic relationships were conducted. For phylogenetic identification of b-TEFAP amplicons, latest available SILVA 16S rRNA gene database was uploaded into mothur and used to classify sequences and OTUs by using a confidence threshold of 80% (bootstrap). All sequences generated in the present study can be accessed through National Center for Biotechnology Information (NCBI) under the bioproject number: PRJNA241494.

### Statistical analyses

Unless otherwise stated, all statistical analyses were performed using PRIMER 6 [60]. In order to analyse the influence of the discriminative environmental parameters on BCC, canonical correspondence analyses (CCA) were performed using the permutest option in the R package VEGAN [61]. The relative abundances of bacterial OTUs were square-transformed, and resemblances between samples were computed by using the Bray-Curtis similarity coefficient [60]. The effect of time, location and oxygen conditions on BCC was tested with one-way analysis of similarities (ANOSIM). In order to establish a relationship between the BCC and the environmental variables analysed, the BIOENV procedure [62] was used by computing Spearman rank correlations for each combination of environmental variables and community composition [63]. We also quantified the relative contribution of several environmental parameters (i.e., Zeu, Zm, temperature, pH, conductivity, DO, turbidity, total alkalinity, BOD_5_, COD, Chl *a*, DOC, TP, SRP, DIN concentrations; see Table 1) as well as bacterial production, phytoplankton species composition on the bacterial community shifts using variation partitioning analyses [64] by means of the varpart function in the VEGAN R package [61]. In addition, a Factorial Correspondence Analysis (FCA), based on the relative abundances of bacterial groups in the three reservoirs at each layer and sampling date, was performed using the ade-4 R package [65].

## Results

### Limnological characteristics of the reservoirs

The analysed reservoirs in the present study evidenced clear differences in their vertical structure of the water column. The three reservoirs presented a consistent thermal stratification from mid-April to mid-September (S2 Fig.). The whole water column remained oxic in La Gileppe reservoir all over the year, in accordance with its oligotrophic status, whereas in the poly-eutrophic reservoir Féronval the hypolimnion was oxygen depleted from beginning of summer to autumn. Ry de Rome reservoir presented hypolimnetic oxygen depletion only shortly in autumn. Conductivity and pH differed widely among reservoirs, in relation with their contrasting mineralisation and alkalinity (Table 1). The average concentrations of inorganic nutrients are also reported in Table 1. Dissolved phosphate concentrations were lower than 5.0 µg-P L^−1^ in the three reservoirs except in the hypolimnion of Féronval reservoir. Total P and DIN concentrations were also much higher in Féronval, in agreement with its reported poly-eutrophic status. DIN was dominated by nitrate in all reservoirs, with ammonium and nitrite often barely detected. The organic carbon content (DOC and TOC) was lowest in Ry de Rome reservoir and highest in La Gileppe reservoir, particularly in autumn samples. DOC and TOC values did not vary substantially with depth. Worth noticing is that BOD_5_ was lowest in La Gileppe reservoir despite its high DOC and TOC, indicating a high proportion of refractory organic carbon (e.g., humic acids), which is also reflected by the low pH and water transparency in La Gileppe.

### Phytoplankton biomass and composition

**Figure 1:**
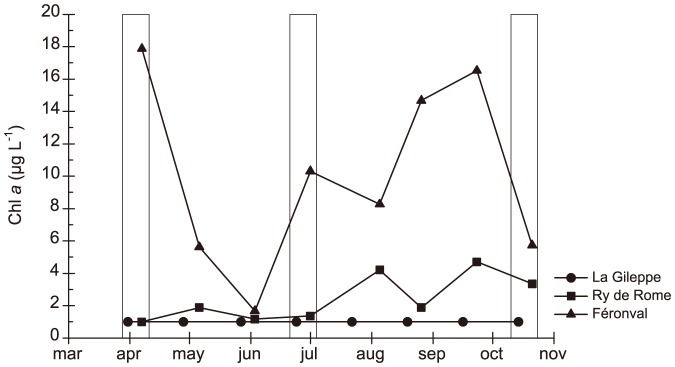
Chlorophyll *a* concentrations. Chlorophyll *a* concentrations (average values from the three analysed water layers) from April to October 2010 measured in La Gileppe (dot), Ry de Rome (square) and Féronval (triangle). Boxes indicate sampling periods for both chemical and BCC analyses.

**Figure 2:**
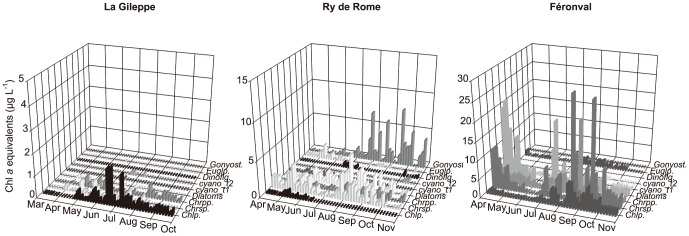
Phytoplantkon community composition. Phytoplantkon community composition over time (from April to October 2010) in the three analysed reservoirs by means of CHEMTAX pigment analyses and expressed as Chlorophyll *a* equivalents (μgr L^−1^).

The temporal variation in Chl *a* (average values of six depths sampled monthly in each reservoir from March to October 2010) highlighted the differences among the analysed reservoirs and periods (Fig. 1). Chl *a* in La Gileppe did not exceed 1.0 µg L^−1^ (Fig. 1), with a phytoplankton assemblage dominated by coccal green algae (*Crucigenia* sp.) and small chrysophytes and cryptomonads (Fig. 2). In Ry de Rome reservoir, Chl *a* ranged between 1.0 and 4.7µg L^−1^ (Fig. 1) and phytoplankton was co-dominated by diatoms (with a *Tabellaria* sp. peak in spring, followed by *Asterionella formosa* and *Nitzschia acicularis* in summer and autumn) and chrysophytes (*Dinobryon* sp.); peaks of *Gonyostomum semen*, a raphidophyte frequently reported in humic lakes, appeared in late summer and autumn (Fig. 2). In this small, stratified and clear reservoir, Chl *a* maxima occurred at 5 m depth (i.e., in the metalimnion). In contrast, in the poly-eutrophic Féronval reservoir, Chl *a* varied between 5.0 and 20.0 µg L^−1^ (Fig. 1), and the assemblage varied greatly over time, with peaks of diatoms (*Nitzschia acicularis, Asterionella formosa* as dominant species), coccal green algae (*Coelastrum microporum*, *Pediastrum* spp., *Scenedesmus* spp., among few others), cyanobacteria (mostly *Aphanizomenon flos-aquae* and *Planktothrix agardhii*), cryptomonads and chrysophytes (Fig. 2).

### Bacterial cell abundances and production

Total cells abundances ranged from 1.0×10^6^ to 1.2×10^7^ cells mL^−1^ with consistent differences among water layers and reservoirs (S3 Fig.). Féronval reservoir presented the highest cell abundances over the analysed periods whereas lower values were recovered for Ry de Rome and La Gileppe reservoirs. Interestingly, the oligotrophic La Gileppe reservoir evidenced higher mean values than the mesotrophic Ry de Rome reservoir during mid-April. In general, higher cell abundances were observed in epi- and metalimnetic samples independently of reservoirs and sampling date, with the exception of spring samples from La Gileppe and autumn ones from Féronval (S3 Fig.).

BP experiments were performed at three depths representing the water layers in stratified conditions. Higher BP values were observed in the poly-eutrophic Féronval reservoir (4.0 and *ca.* 40.0 times higher) in comparison to the lower ones obtained in the meso- and oligotrophic reservoirs (S3 Fig.). In Lake Féronval, BP clearly decreased from epilimnion to hypolimnion in April and July, whereas BP was fairly constant and lower in October. Interestingly, the lowest BP values were obtained in samples from the mesotrophic reservoir, especially in samples from epilimnetic waters (S3 Fig.).

### Pyrosequencing data overview

#### Richness and diversity estimates

For all samples analysed by pyrosequencing, the quality criteria applied for reduction of sequencing noise, chimeric sequences and presence of contaminants omitted less than 10.0% of total sequences (data not shown). The curated (i.e., chimera-free and without contaminant sequences) 77815 bacterial 16S rRNA gene sequences (range: 995 – 4883 per sample) resulted in 4031 bacterial OTUs (0.03 cut-off; range: 118 – 654 OTUs per sample; see details in Table 2), which represented most of the richness in the analysed samples, thus supporting obtained coverage values (89.7% – 97.7% coverage estimates). Rarefaction curves (data not shown) reached saturation for all analysed samples. For all the samples analysed, values of richness (Chao1) and diversity (Shannon) estimators were higher in Ry de Rome and Féronval reservoirs than in La Gileppe (Table 2). Furthermore, rank abundance curves for each reservoir (S4 Fig.) showed dominance of one single OTU identified as a member of the *Actinobacteria* (hgcI_clade) representing between 9.9% and 38.6% of sequences retrieved from each reservoir when all samples of a given reservoir were pooled together (data not shown). Moreover, the second and third most abundant OTUs were affiliated to members of *Actinobacteria* (hcgI_clade*)*, *Alphaproteobacteria* (SAR11), *Betaproteobacteria* (*Polynucleobacter*), and *Verrucomicrobia* (vadin HA64) clades with relative abundances ranging from 6.5% to 12.4% of retrieved sequences for each reservoir (data not shown).

**Table 2:** Observed bacterial richness and diversity estimates based on OTUs for La Gileppe, Ry de Rome, and Féronval reservoirs.

					Richness^d^	Diversity^d^
Reservoir^a^	WC^b^	Season^c^	Filtered reads	OTUs	Chao1 (± sd)	Shannon (± sd)
G	E	Apr	2606	203	445±97	2.9±0.1
G	E	Jul	2149	118	193±37	1.5±0.1
G	E	Oct	2938	131	210±37	2.1±0.1
G	M	Apr	2892	299	774±167	3.3±0.1
G	M	Jul	2819	234	444±82	2.4±0.1
G	M	Oct	2819	294	671±134	3.1±0.1
G	H	Apr	2058	255	581±117	3.3±0.1
G	H	Jul	1559	250	594±125	3.6±0.1
G	H	Oct	2033	303	507±72	4.1±019
						
RR	E	Apr	2952	340	908±201	3.9±0.1
RR	E	Jul	3211	235	590±140	2.9±0.1
RR	E	Oct	995	178	306±54	3.8±0.1
RR	M	Apr	3621	351	721±123	3.9±0.1
RR	M	Jul	3724	296	627±121	3.6±0.1
RR	M	Oct	1328	243	517±104	4.0±0.1
RR	H	Apr	3575	363	667±102	4.1±0.1
RR	H	Jul	4239	382	769±126	3.8±0.1
RR	H	Oct	1964	321	497±62	4.6±0.1
						
F	E	Apr	1782	190	289±43	3.7±0.1
F	E	Jul	3660	286	501±80	3.7±0.1
F	E	Oct	3117	381	678±98	4.2±0.1
F	M	Apr	3012	289	559±101	3.9±0.1
F	M	Jul	3053	451	793±101	4.5±0.1
F	M	Oct	4300	555	910±98	4.7±0.1
F	H	Apr	2833	408	830±134	4.6±0.1
F	H	Jul	4883	371	673±99	3.5±0.1
F	H	Oct	3693	654	1278±156	5.0±0.1
						

a , G, Gileppe; RR, Ry de Rome; F, Féronval.

b , Water layer: E, epilimnion; M, metalimnion; H, hypolimnion.

c , Apr, April 2010; Jul, July 2010; Oct, October 2010.

d , data for OTU level (0.03 cut-off).

#### Bacterial Community Composition

**Figure 3:**
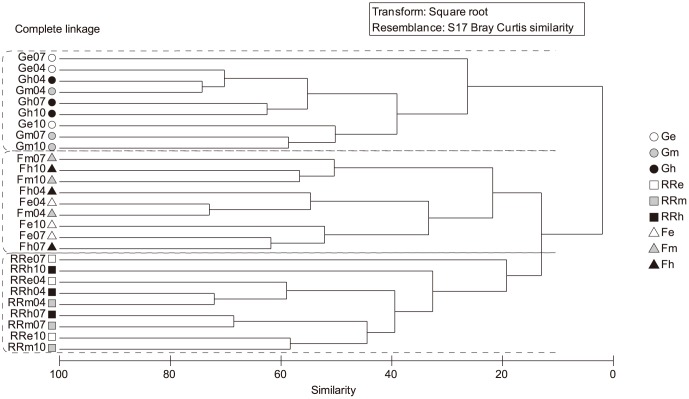
Clustering analyses. Hierarchical clustering analyses of Bray-Curtis similarities of the community composition at OTU level (0.03 cut-off) in the three reservoirs. Legend: G, La Gileppe (dots); RR, Ry de Rome (square); F, Féronval (triangles); e, epilimnion (white); m, metalimnion (grey); h, hypolimnion (black); 04, April 2010; 07, July 2010; 10, October 2010.

Hierarchical clustering based on the relative abundances of bacterial OTUs showed a clear structuring effect of water sample origin, resulting in three major clusters related to the analysed reservoirs (Fig. 3). Accordingly, samples from La Gileppe reservoir were significantly different and distinct from those of Ry de Rome and Féronval reservoirs (Fig. 3; p<0.01) as was also evidenced by ANOSIM analyses (Table 3). When comparing the BCC retrieved in the three reservoirs at different seasons, ANOSIM analyses showed differences in the BCC between April and July and between April and October, but not between July and October. However, clustering results presented in Fig. 3 clearly show that the origin of the samples (i.e., reservoir in which the sample was collected) clearly dominated the seasonal effect.

**Table 3:** ANOSIM significance values based on OTUs (0.03 cut-off value).

Test for differences between sampling locations Across all layer groups	R	*p*
Global test	0.989	0.001
Pairwise test		
La Gileppe – Ry de Rome	1.000	0.002
Féronval – Ry de Rome	0.975	0.002
Féronval – La Gileppe	1.000	0.001

Test for differences between water layers Across all lake groups	R	*p*
Global test	−0.037	0.630
Pairwise test		
Epilimnion – Metalimnion	−0.012	0.550
Epilimnion – Hypolimnion	0.160	0.097
Hypolimnion – Metalimnion	−0.210	0.930

Test for differences between sampling dates Across all lake groups	R	*p*
Global test	0.503	0.010
Pairwise test		
April – July	0.704	0.001
April – October	0.617	0.004
July – October	0.259	0.090

**Figure 4:**
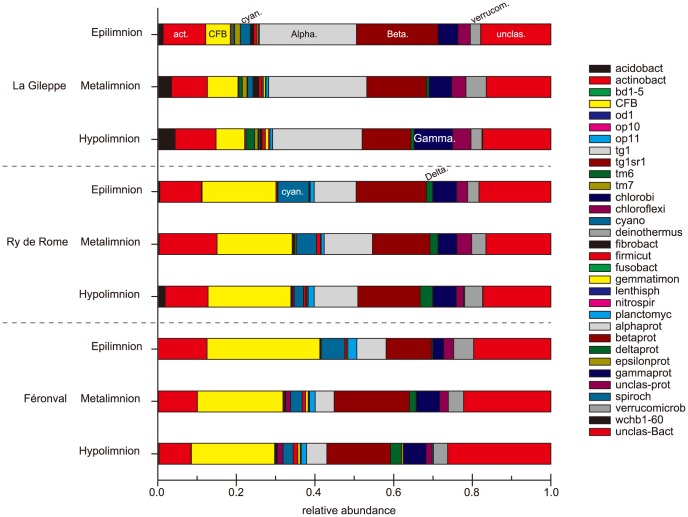
Bacterial community composition. Relative proportion of bacterial retrieved groups (by b-TEFAP) at OTU level (0.03 cut-off) in the water layers of the three analysed reservoirs.

At phylum level, the three analysed reservoirs presented similar BCC with the dominance of few but typical freshwater bacterial groups (Fig. 4 and S1 Table) even though conspicuous variations in their relative abundance were also observed. Taxonomic affiliations evidenced *Betaproteobacteria* (range: 10.1% – 26.6%), *CFB* (range: 5.4% – 32.9%), *Alphaproteobacteria* (range: 3.5% – 32.0%), and *Actinobacteria* (range: 5.6% – 22.6%) as the four most abundant bacterial groups recovered in each reservoir (from first to fourth). The relative abundance of *CFB*-related OTUs was higher in Ry de Rome (deep waters) and Féronval (epilimnetic waters) reservoirs, whereas relative abundance of *Alphaproteobacteria* showed an opposite pattern (higher recovery in La Gileppe reservoir). *Betaproteobacteira* presented higher relative abundance in the epilimnetic waters of the less productive reservoirs (i.e., oligotrophic and mesotrophic) mainly due to *Burkholderiales* and unclassified betaproteobacterial OTUs contribution (S1 Table), which contrasted with their lower recovery in the deep waters of the poly-eutrophic reservoir (S1 Table). In turn, *Actinobacteria*-related OTUs showed similar values all over the three water layers, except for markedly higher values in metalimnetic waters of Ry de Rome reservoir and in the epilimnion of Féronval reservoir. Furthermore, unclassified bacterial OTUs (range: 8.4% – 30.1% of retrieved amplicons) also represented a large fraction in the analysed reservoirs, especially in meta- and hypolimnetic waters of Féronval reservoir. Some less abundant groups also exhibited a contrasted pattern. For instance, *Acidobacteria* were slightly more abundant in La Gileppe reservoir (average for all depths and sampling dates *ca*. 2.6% of the BCC) than in Ry de Rome (*ca*. 0.7%) and Féronval (*ca*. 0.2%) reservoirs. In contrast, an inverse pattern for *Deltaproteobacteria* was observed (average values of *ca*. 0.3%, 1.9%, and 1.0% for La Gileppe, Ry de Rome and Féronval; respectively).

In La Gileppe reservoir, the three water layers showed similar BCC (Fig. 4 and S1 Table) all over the three seasons, with dominance of *Alpha-*, *Betaproteobacteria*, *Actinobacteria* and unclassified OTUs. *Alphaproteobacteria* represented the main bacterial group in the oligotrophic reservoir although a lower relative abundance was observed in epilimnetic waters during mid-October. Moreover, slight changes in the hypolimnion were detected, including a higher relative contribution of *Gammaproteobacteria* (range: 7.6% – 10.2%) in comparison with the other retrieved groups. Furthermore, inverse patterns between *Betaproteobacteria* (decreasing values from epi- to hypolimentic waters ranging from 25.4% to 11.5%) and *Gammaproteobacteria* (increasing values from epi- to hypolimnetic waters; range: 4.2% – 10.2%) were also observed. No clear trend was observed for other typical freshwater groups like *Verrucomicrobia* and *Acidobacteria* (only detected in La Gileppe), even though slightly higher values were observed in all samples from meta- and hypolimnetic waters (range: 2.0% – 6.4% and 2.1% – 4.9%, respectively).

In the mesotrophic reservoir, the three water layers showed a BCC dominated by *Betaproteobacteria*, *CFB*, *Actinobacteria* and *Alphaproteobacteria*. No clear seasonal trend could be observed. Higher values of *CFB* (range: 13.5% – 25.2%) were obtained in hypolimnetic waters, whereas a higher relative abundance of *Actinobacteria* was observed in metalimnetic (range: 17.1% – 22.6%) samples. Other minor groups like *Cyanobacteria* (range: 2.0% – 11.5%) were also recovered from epi- and metalimnetic waters in Ry de Rome reservoir with higher relative abundance in mid-July samples (11.5% and 7.7% for epi- and metalimentic water samples, respectively). Slightly increasing relative abundance values with depth were observed for *Verrucomicrobia* in mid-July samples, but never exceeding 9.0% of the total BCC. *Deltaproteobacteria* evidenced higher relative abundance values in the hypolimnion, but never exceeded *ca*. 6.0% of total BCC.

*CFB*, *Actinobacteria*, *Betaproteobacteria*, *Alphaproteobacteria* and unclassified bacterial OTUs were also the main members of the BCC of the poly-eutrophic reservoir, as for the other studied reservoirs, but Lake Féronval harboured the highest contribution of *CFB* and the lowest one of *Alphaproteobacteria*. A slight decreasing trend of *CFB* relative abundance with depth and sampling period (from April to October) was observed. This trend was opposite for unclassified bacterial OTUs. As for *Actinobacteria*, a marked decreasing pattern with respect to depth (from epilimnion to hypolimnion) and period (from April to October) was observed. In the case of *Betaproteobacteria*, higher relative abundances were observed in meta- and hypolimnetic waters at the three seasons. *Alphaproteobacteria* represented a minor fraction of Féronval reservoir BCC with relative abundance values ranging from 3.5% to 7.3%. *Delta*- and *Gammaproteobacteria* (*Methylococcales* in poly-eutrophic reservoir Féronval and *Legionellaceae* in La Gileppe and Ry de Rome) showed increasing relative abundances with depth.

#### Overlapping microbial communities

**Figure 5:**
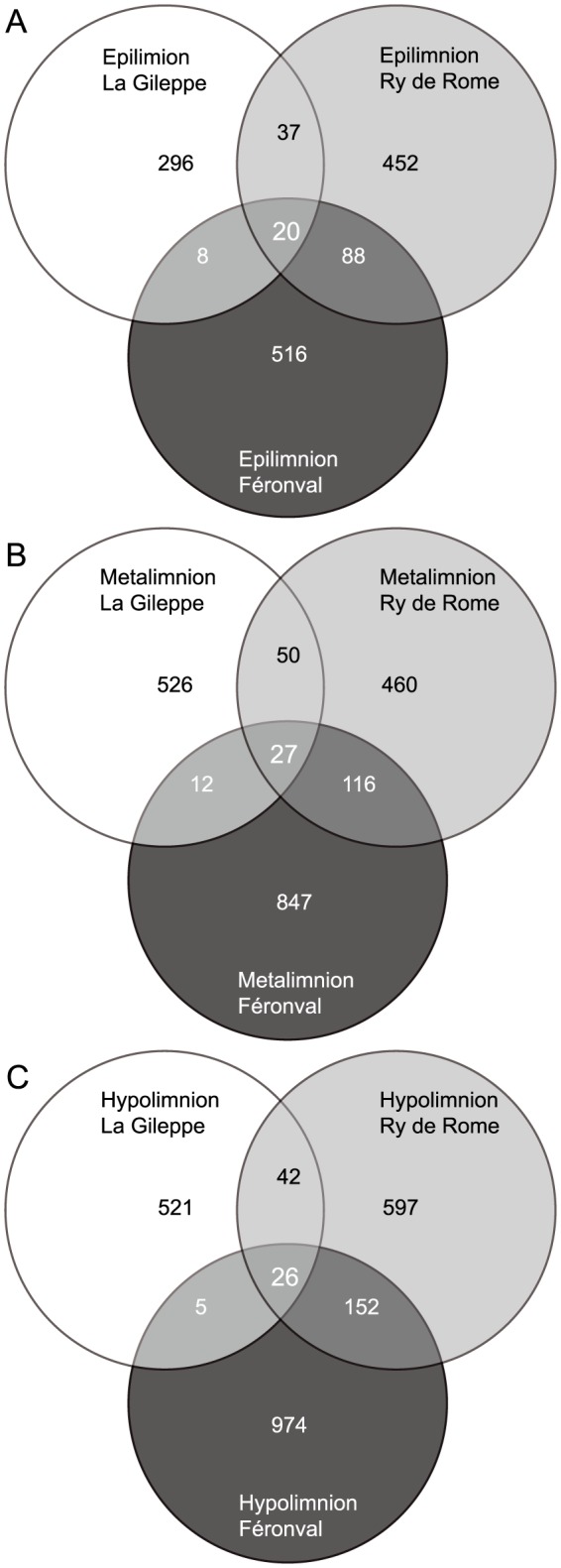
Shared bacterial community. Venn diagrams highlighting shared OTUs for each analysed water compartment (**A**, epilimnion; **B**, metalimion; and **C**, hypolimnion) and reservoir (i.e., La Gileppe, Ry de Rome and Féronval).

Analyses of shared OTUs among the three reservoirs and water compartments were conducted by considering each environment as a whole (data not shown). In this sense, only 1.5% (59) of the OTUs were shared among the three freshwater reservoirs thus representing the core bacterial community of the analysed reservoirs microbiome (data not shown). Of those 59 OTUs, nine were highly abundant (1.4% – 11.6% of the reads) and were assigned to hgcl-clade (*Actinobacteria*), *Methylophilaceae* and *Burkholderiales* (*Betaproteobacteria*), and *Chitinophagaceae* (genera *Sediminibacterium* of *CFB*) families and to the uncultured vadinHA64 (*Opitutae* class of *Verrucomicrobia*) group. Besides, 8.75% of retrieved OTUs were present at least in two microbiomes (i.e., two reservoirs). A higher number of OTUs (233; 5.78%) was shared between the mesotrophic and the poly-eutrophic reservoirs whereas only 104 OTUs (2.58%) were shared between those reservoirs within the lower nutrient content range (data not shown). Interestingly, those reservoirs with the most different features only shared 16 OTUs (0.40%, data not shown). Besides, the highest number of unique OTUs (1613, 40.0%) was found in the poly-eutrophic reservoir whereas the oligotrophic, acid reservoir contained the lowest number (971 unique OTUs, 24.1%). Highly abundant and unique OTUs in La Gileppe reservoir belonged to *Acetobacteraceae*, *Betaproteobacteria*, *Burkholderiales*, *Alphaproteobacteria*, hgcl-clade and *Acidobacteria*, whereas in Ry de Rome reservoir belonged to *Flavobacterium*, hgcl-clade and *Comamonadaceae*. In turn, in the poly-eutrophic Féronval reservoir, abundant OTUs were more equally distributed between the *Actinomycetales*, *Flavobacterium*, *Polynucleobacter*, *Methylococcales*, hgcl-clade, and *Planctomycetales*. Venn diagrams were constructed in order to analyse the shared microbiome in each water compartment of the three reservoirs (Fig. 5A – C) clearly showing an increase in the number of OTUs with depth. Higher numbers of unique and shared OTUs were observed in relation to increasing nutrient loading. In this sense, La Gileppe contained less unique OTUs than Féronval reservoir and at the same time La Gileppe and Féronval shared less OTUs than Ry de Rome and Féronval, when comparing the three epilimnia and hypolimnia between them (Fig. 5A and C). However and due to the transition status that could characterize metalimnetic waters, Ry de Rome presented the lower number of exclusive OTUs (22.6%).

### Environmental factors driving BCC

**Figure 6:**
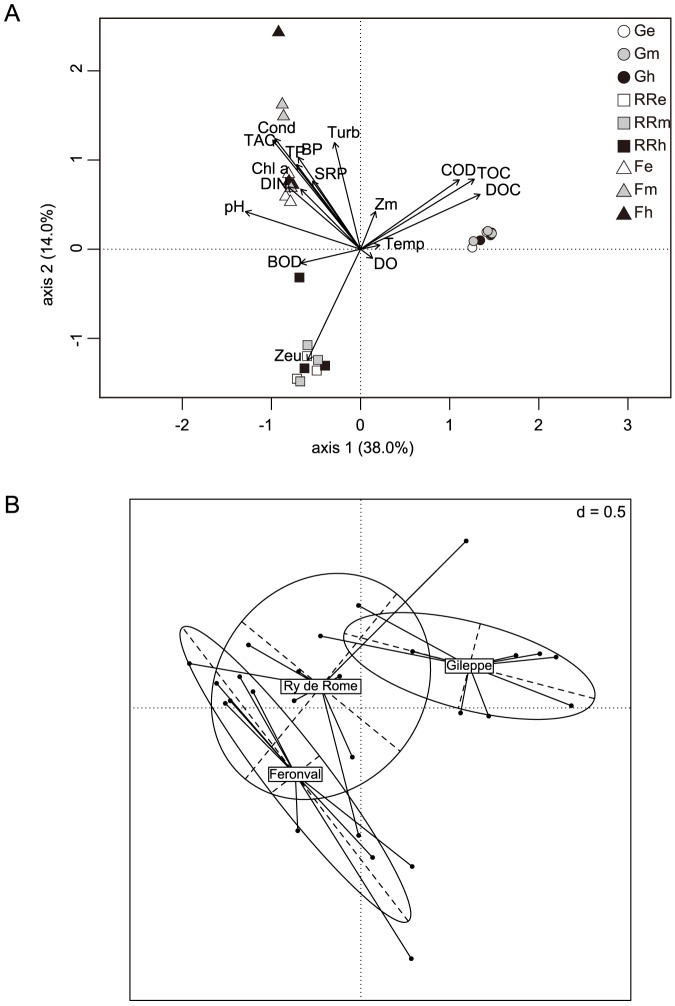
Canonical and Factorial correlation analyses. Canonical correlation analyses (**A**) and factorial correspondence analysis (**B**) of bacterial communities based on relative abundance at OTU level (0.03 cut-off) and environmental parameters in the analysed reservoirs and all sampling dates. Legend: G, La Gileppe (dots); RR, Ry de Rome (square); F, Féronval (triangles); e, epilimnion (white); m, metalimnion (grey); h, hypolimnion (black); 04, April 2010; 07, July 2010; 10, October 2010.

Statistical analyses of the analysed environmental parameters by means of CCA showed a clear segregation among lakes (permutest, F = 2.19, p = 0.001). In particular, samples from La Gileppe reservoir formed a cluster well separated from those of the two other reservoirs in the first axis; as Ry de Rome and Féronval reservoirs grouped separately mainly along the second axis with a smaller covariance (Fig. 6A and B). The DOC, TOC and COD were shown to be associated with BCC in La Gileppe, whereas BCC in Féronval was correlated with multiple parameters such as pH, Chl *a*, TP, BP and total alkalinity. The effect of environmental parameters on BCC was confirmed by Mantel tests (BIO-ENV, Table 4), where DOC and TOC together with pH and TA were evidenced as the strongest factors structuring BCC (Fig. 6B).

**Table 4:** Results from BIO-ENV analyses of bacterial community composition and environmental parameters.

Variables	ρ
DOC	0.669
pH	0.582
TOC	0.572
TAC	0.526
pH, TAC, DOC, TOC	0.722
pH, TAC, DOC	0.764
pH, Cond, DOC, TOC	0.763

The Spearman rank ρ values indicate rank correlation between the matrix of bacterial community composition similarity and the similarity matrices from environmental variables. Only significant *p* values are shown.

Furthermore, the abundance profile of each OTU was correlated with each measured environmental parameter and those OTUs that were significantly linked to any environmental parameter were identified (Pearson correlation ρ>0.85, p<0.05, S2 Table). In this sense, a strong correlation between Chl *a* and OTUs related to *Flavobacteriales*, *Comamonadaceae*, *Cyanobacteria* and hgcl-clade (*Actinobacteria*) were observed; whereas, positive correlations between DIN and *Cyanobacteria* were also observed, especially in Féronval reservoir.

## Discussion

In the present study, three distinct freshwater reservoirs were analysed covering a wide range of physical and chemical contrasting conditions (Table 1) resulting in distinct phytoplankton (Fig. 2) and bacterioplankton (Fig. 5 and S1 Table) community compositions. The bacterial diversity identified in the three freshwater reservoirs further extends the presence and dominance of few bacterial groups previously detected in epilimnetic waters of natural freshwater lakes [34]. In our study, we examined the recovery of these cosmopolitan bacterial groups according to water physical and chemical properties, water layers and seasons in man-constructed lakes.

In concordance with previous works on freshwater lakes [21], [34], the three reservoirs presented a typical freshwater bacterial community with a core microbiome mainly composed by *Actinobacteria* (hgcI_clade), *Betaproteobacteria* (*Rhodoferax* and *Polynucleobacter*) and *Verrucomicrobia* (vadinHA64) groups. In this sense and in agreement with their stable [66] and cosmopolitan [34] behaviour, members of *Actinobacteria* have been detected through time and space in all three analysed water bodies (relative abundance never exceeding 20.0%, Fig. 5 and S1 Table). A possible explanation for the widespread detection of *Actinobacteria* could be the reduction of grazing losses due to their reduced cell size and cell wall type [34], [67]–[69]. Accordingly, no clear trend for *Actinobacteria* could be detected depending on reservoir trophic status. However, lower relative abundance of *Actinobacteria* was observed in the meta- and hypolimnion than in the epilimnion of the studied poly-eutrophic reservoir as observed in several lakes of contrasting limnological characteristics [70], [71].

Data from the present study showed high relative abundance of *CFB* in the meso- and poly-eutrophic reservoirs, agreeing with current knowledge on *CFB* showing that this bacterial group was mainly found in environments containing high autochthonous organic carbon [72], [73]. These two reservoirs are prone to phytoplankton blooms, with blooms of *Gonyostomum semen* in the mesotrophic Ry de Rome reservoir and a rich community of diatoms, cryptophytes and chrysophytes in the poly-eutrophic Féronval reservoir. Accordingly, a low contribution of *CFB* to the BCC was observed in the oligotrophic La Gileppe reservoir, where DOC is mostly allochthonous, supporting previous evidences of low relative contribution of *CFB* in lakes characterized by low primary productivity [72]–[74].

*Alphaproteobacteria* have been described to be dominant in oligotrophic and in acid humic rich environments [75]. In the oligotrophic reservoir studied, a higher relative abundance of *Rhizobiales* and *Rhodospirillales* was obtained, whereas lower relative abundances were observed in the meso- and poly-eutrophic reservoirs, with much lower content of humic acids. However, our data could not establish a clear link between *Alphaproteobacteria* and *Cyanobacteria* as previously evidenced for marine and freshwater environments [76]. *Betaproteobacteria* usually represent the most abundant bacteria (up to 60 – 70% of total cells numbers) inhabiting lacustrine epilimnetic waters [34], with BetI and BetII groups being responsible for the overall *Betaproteobacteria* dominance in freshwater habitats [77]. BetI tribe (e.g., BAL47, *Rhodoferax*, GSK and *Limnohabitants* clades) are known to prefer algal-derived DOC [20] while members of the BetII tribe (e.g., *Polynucleobacter*) mostly consume photo-oxidised products from humic acids [78], [79]. Data retrieved in the present study evidence similar relative abundance of *Betaproteobacteria* in the three studied reservoirs. However, a dominance of *Burkholderia*-related OTUs was observed in the epilimia of La Gileppe and Ry de Rome while a dominance of *Rhodocyclales*-related OTUs was observed in meta- and hypolimnetic waters of Féronval reservoir in relation to its high nitrogen content (Table 1 and Fig. 6A). Furthermore, *Gammaproteobacteria* were not particularly abundant in the three studied reservoirs as was also reported for freshwater lakes [31] even if culture studies [39], [80] suggest better growth of this group under N- and P-enriched media. Our results, on the contrary, evidenced a slightly higher relative abundance of *Gammaproteobacteria* in the hypolimnion of La Gileppe reservoir (Fig. 4), which has lower TP content than the poly-eutrophic Féronval reservoir (Table 1). Furthermore, the most recovered *Gammaproteobacteria* (*Methylococcales,* representing up to 50% of all *Gammaproteobacteria*) were more abundant in the poly-eutrophic Féronval reservoir, which could indicate putative methane oxidation processes in this water body.

Another cosmopolitan bacterial inhabitant of freshwater lakes are *Verrucomicrobia*, which showed a wide geographical distribution [34]. This bacterial group was recovered from the three reservoirs studied supporting the idea of *Verrucomicrobia* as a bacterial group possessing a wide variety of metabolic strategies [81].

The present study reveals significant differences at BCC level per reservoir. In this sense, the BCC of the mesotrophic Ry de Rome reservoir was more similar to the BCC of the poly-eutrophic reservoir Féronval than to the oligotrophic one (i.e., La Gileppe; Fig. 3). By contrast, a fingerprinting analysis conducted in several Portuguese water bodies, including reservoirs, lakes and rivers, evidenced more similarities among oligo- and mesotrophic water bodies than with respect to eutrophic ones [82]. The observed BCC similarities in the present study between Féronval and Ry de Rome reservoirs might be related to the origin of their organic carbon (i.e., autochthonous) and to phytoplankton community composition. Several studies evidenced that freshwater lakes from the same catchment area (as the case of Féronval and Ry de Rome) were more similar in terms of BCC than isolated and not-connected lakes, in which these similarities could not be related to water transport of bacterial cells [83], [84]. Interestingly, the closest reservoirs in terms of distance also showed strong differences with respect to BP, cell abundances and nutrient content (S3 Fig. and Table 1). The differences mentioned above might also contribute to explain the differences observed in the BCC in relation to those heterotrophic microbes like *CFB* and *Alpha-* and *Betaproteobacteria* (Fig. 4 and S1 Table). BP values obtained in the present study were in agreement with previously reported values for temperate lakes [85].

In contrast with other studies carried out in freshwater lakes that evidenced a strong effect of depth in BCC structure [15], [86], no clear vertical stratification of the BCC within the water column was observed. The similarity among water layers might result from the fact that present microbial groups have broad metabolic capabilities, and were able to develop in both oxic and illuminated epilimnetic waters as well as in anoxic hypolimna. Besides, reservoirs strongly differ from natural lakes in their shorter water residence time and in the fact that they present a longitudinal gradient from their inlet, where river-like conditions prevail, to the dam, where lake-like conditions develop. Accordingly, plankton communities differ along this gradient [87], [88] and it is likely that BCC in reservoirs depends on inputs from the inflowing rivers thus reacting more to the variations along the longitudinal gradients than to the variations among water layers during the stratification period.

The statistical analyses revealed that organic carbon (either total or dissolved) and its origin together with pH and alkalinity exert the strongest segregation effect on the BCC in the studied reservoirs (Fig. 6). The seasonal succession of the BCC observed in the present study was associated with changes in key physical and chemical parameters and also to changes in phytoplankton biomass and community composition, which might reflect interactions between phyto- and bacterioplankton communities. Such correlations between BCC and phytoplankton community were also previously observed across lakes and seasons [89], [90]. Nutrient content in freshwater environments has long been seen as an important environmental factor controlling planktonic community composition [13], [19] as revealed in the present study where differences in both phyto- and bacterioplankton community composition level according to nutrient status (i.e., organic carbon content) were observed but also influenced by pH (understood as a proxy for humic acid) and alkalinity (Fig. 6). Furthermore, other parameters like presence of aquatic vegetation, zooplankton community composition, and viruses might also exert some influence in BCC in the analysed reservoirs [3], [91], [92].

## Conclusions

The present study evidenced differences in terms of physical and chemical data and phytoplankton community composition in the three freshwater reservoirs, whereas they harboured a core microbiome composed of cosmopolitan bacterial groups (*Actinobacteria* (hgcI_clade), *Betaproteobacteria* (*Rhodoferax* and *Polynucleobacter*) and *Verrucomicrobia* (vadinHA64)) as recently stated for the epilimnion of freshwater lakes [34]. The dissimilarities observed in the BCC of the analysed reservoirs mainly resulted from differences in the typology of organic carbon content (i.e., La Gileppe reservoir is rich in allochthonous organic carbon). As for freshwater lakes, data presented here for reservoirs also evidenced strong relationships between trophic status, phytoplankton community and productivity. Future studies might focus on analysing the DOC fluxes, the archaeal counterparts, as well as the bacterial grazing and lysis due to viruses in the reservoirs in order to decipher the potential role in nutrients cycling of the microbial communities present.

## Supporting Information

S1 Fig **Sample locations.** Map of Belgium showing the location of analysed reservoirs (G, for La Gileppe, RR, for Ry de Rome and F, for Féronval) described in this study.
(PDF)

S2 Fig **Temperature depth profiles.** Vertical depth profiles of temperature over time in the three analysed reservoirs (**A**, La Gileppe; **B**, Ry de Rome; and **C**, Féronval) during 2010.
(PDF)

S3 Fig **Bacterial production and abundances.** Bacterial production (BP; bars) and bacterial abundances (DAPI stained cells; dots) in the epilimnion (E), metalimnion (M) and hypolimnion (H) of La Gileppe, Ry de Rome and Féronval reservoirs. Legend: Black symbols correspond to April 2010 samples, white symbols correspond to July 2010 samples and grey symbols correspond to October 2010 samples.
(PDF)

S4 Fig **Rank abundance.** Rank abundance plots of retrieved OTUs (0.03 cut-off) from La Gileppe (black dots), Ry de Rome (grey dots) and Féronval (red dots) reservoirs.
(PDF)

S1 Table **Bacterial diversity.** Bacterial groups retrieved after bTEFAP from the three analysed reservoirs (full depths).
(XLSX)

S2 Table **Pearson correlations.** Pearson correlation between OTUs (0.03 cut-off level) and environmental factors (ρ>0.85, p<0.05).
(XLSX)
