# Supplementary figures and images for: Bacterial Community Composition in Three Freshwater Reservoirs of Different Alkalinity and Trophic Status
Source: PLoS One. 2014 Dec 26;9(12):e116145. doi: 10.1371/journal.pone.0116145 (PMC4277477; doi:10.1371/journal.pone.0116145)

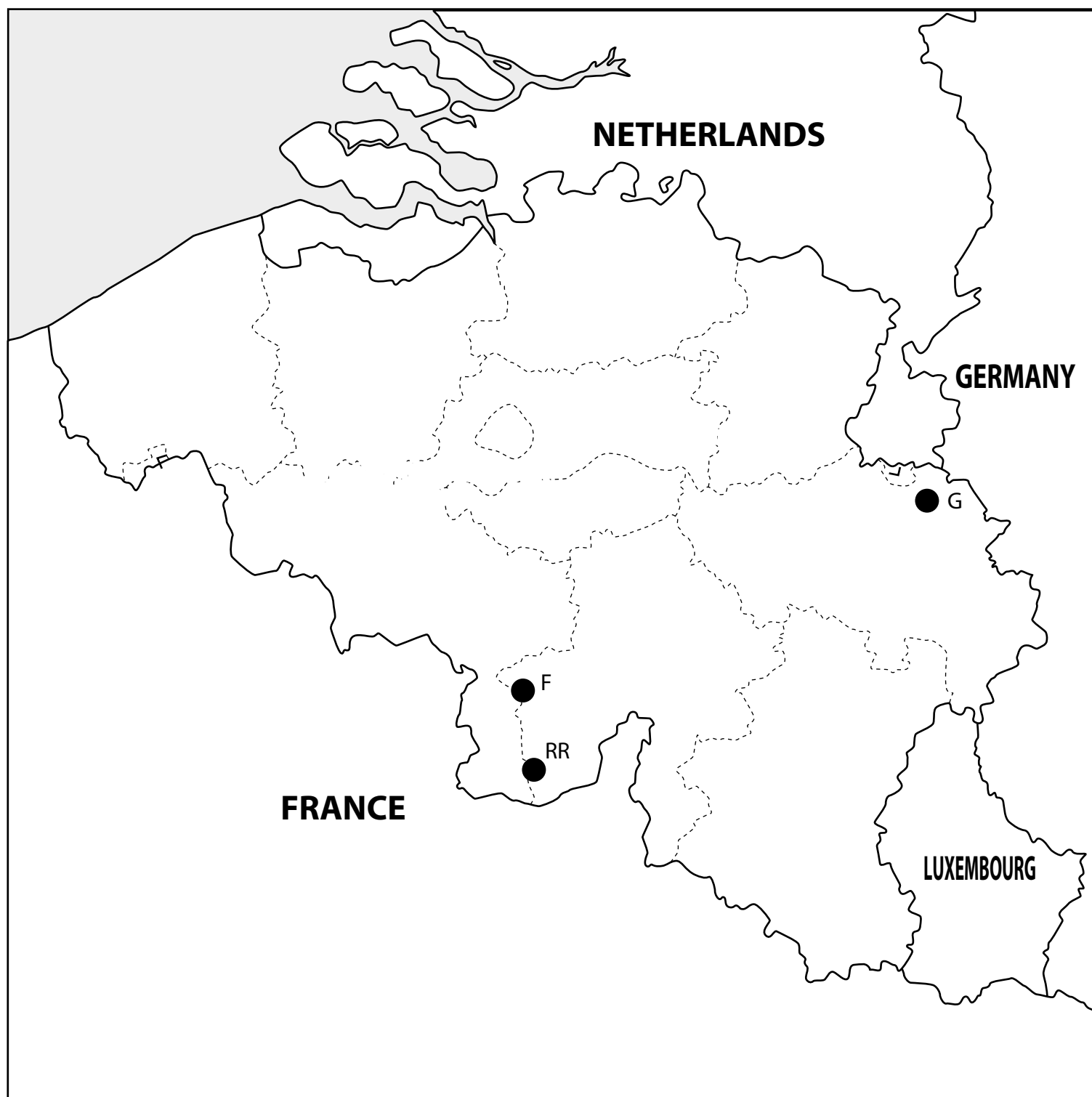

**Figure S1**

Supplement: S1 Fig — Sample locations. Map of Belgium showing the location of analysed reservoirs (G, for La Gileppe, RR, for Ry de Rome and F, for Féronval) described in this study. (PDF) [file pone.0116145.s001.pdf]

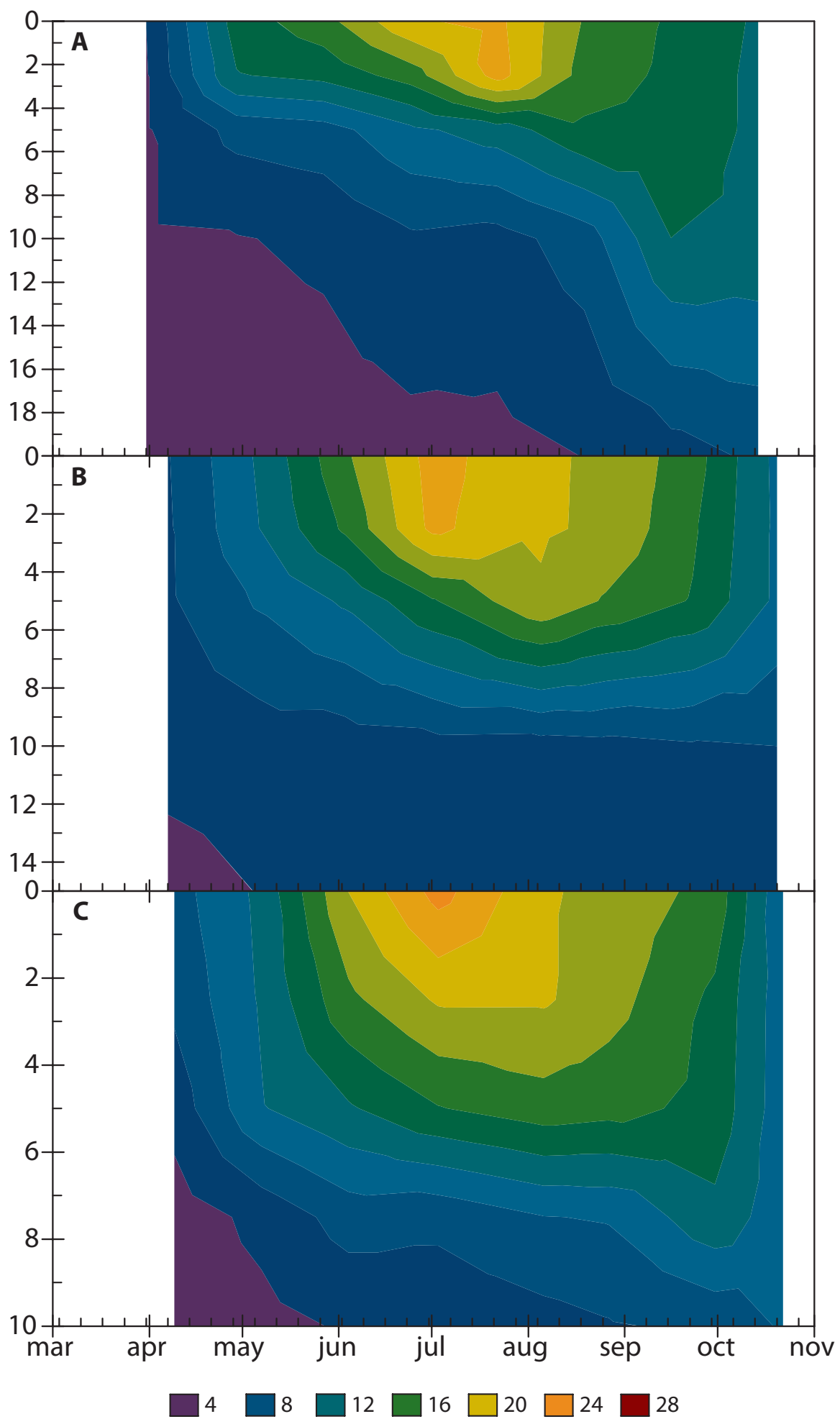

**Figure S2**

Supplement: S2 Fig — Temperature depth profiles. Vertical depth profiles of temperature over time in the three analysed reservoirs (A, La Gileppe; B, Ry de Rome; and C, Féronval) during 2010. (PDF) [file pone.0116145.s002.pdf]

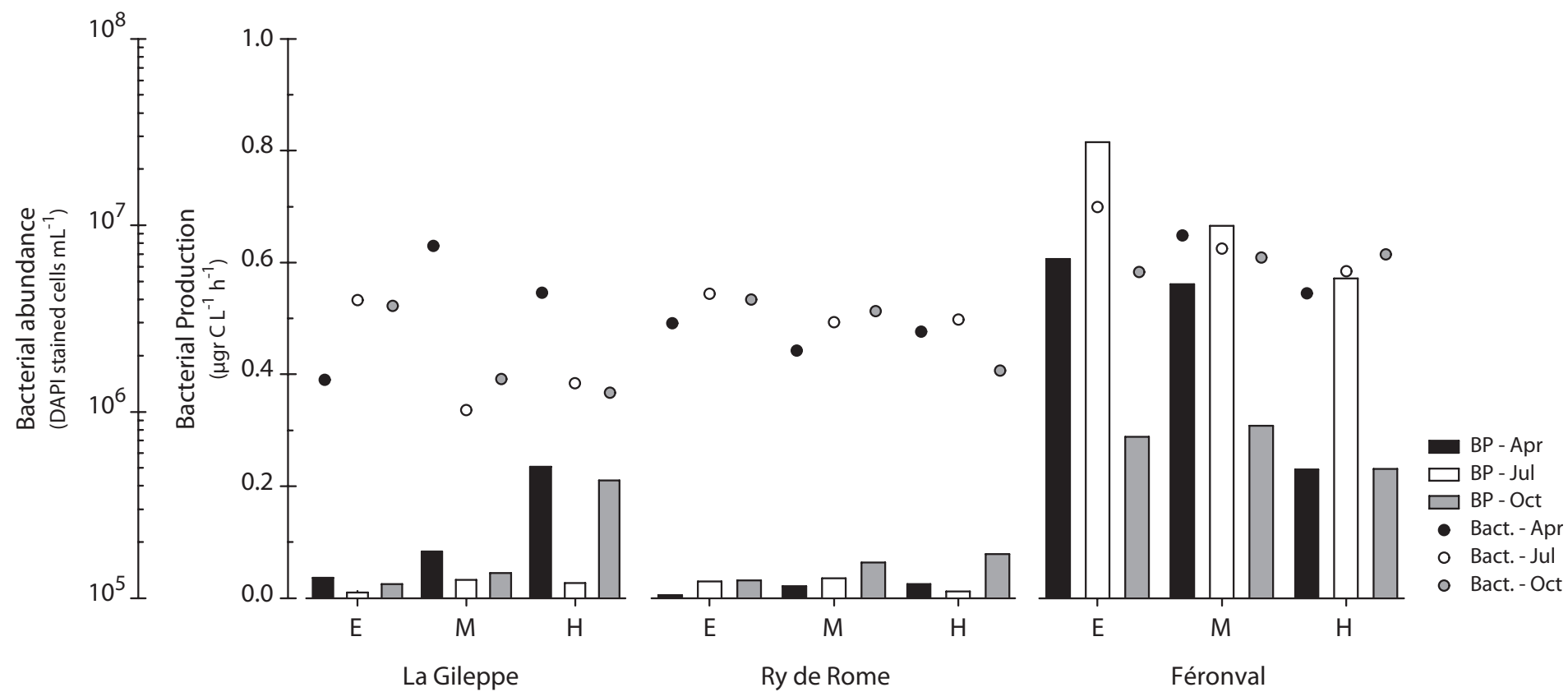

Figure S3

Supplement: S3 Fig — Bacterial production and abundances. Bacterial production (BP; bars) and bacterial abundances (DAPI stained cells; dots) in the epilimnion (E), metalimnion (M) and hypolimnion (H) of La Gileppe, Ry de Rome and Féronval reservoirs. Legend: Black symbols correspond to April 2010 samples, white symbols correspond to July 2010 samples and grey symbols correspond to October 2010 samples. (PDF) [file pone.0116145.s003.pdf]

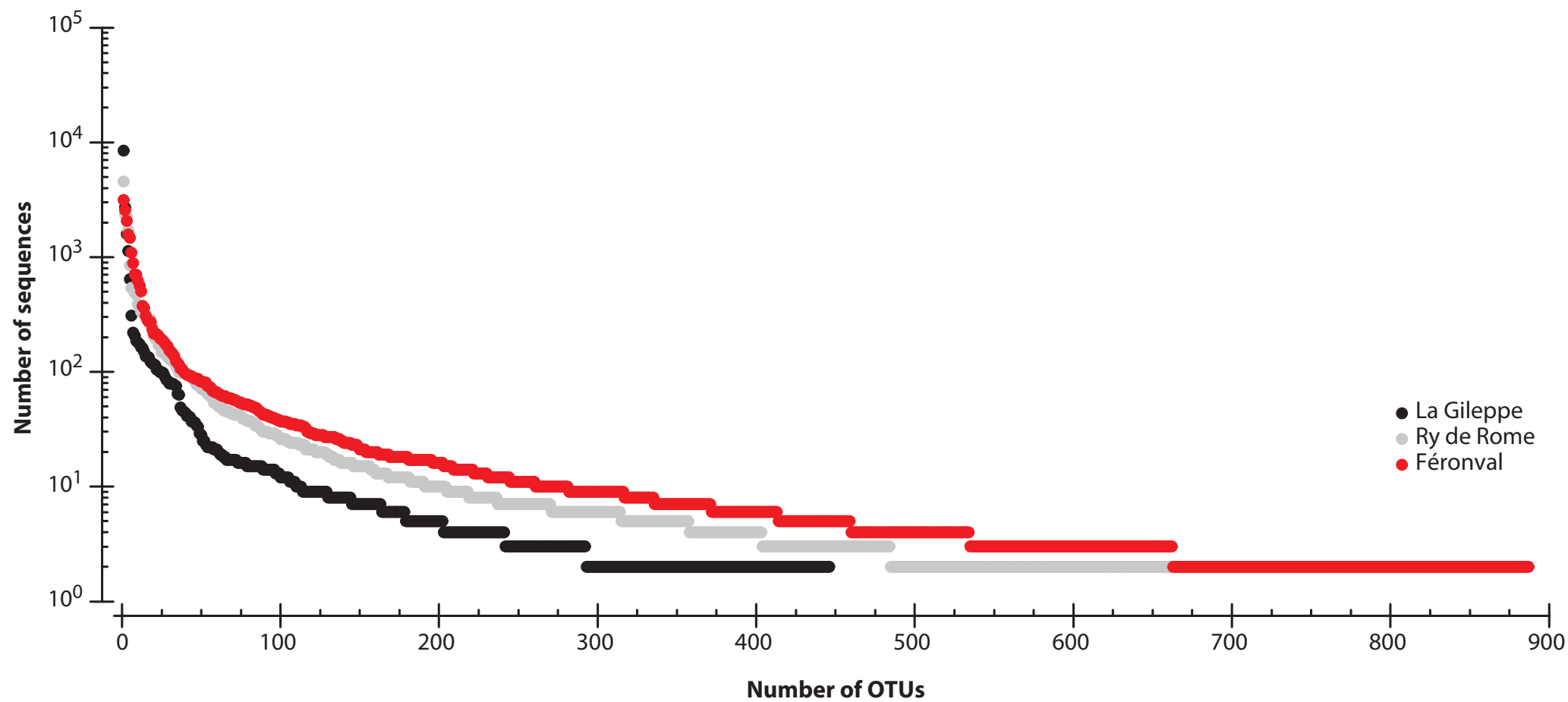

Figure S4

Supplement: S4 Fig — Rank abundance. Rank abundance plots of retrieved OTUs (0.03 cut-off) from La Gileppe (black dots), Ry de Rome (grey dots) and Féronval (red dots) reservoirs. (PDF) [file pone.0116145.s004.pdf]
